# Genetic predisposition to diabetes and risk of stroke in a Native Hawaiian population

**DOI:** 10.1007/s40200-026-01940-5

**Published:** 2026-05-07

**Authors:** Stacy C. Brown, Cameron P. Both, Mālialani Kanaʻiaupuni, Leah Dowsett, Keolu Fox, Kazuma Nakagawa, Mariana Gerschenson, Guido J. Falcone

**Affiliations:** 1 University of Hawaiʻi at Mānoa, Honolulu, HI USA; 2 The Queen’s Medical Center, Neuroscience Institute, Honolulu, HI USA; 3 UMass Chan Medical School, Worcester, MA USA; 4 University of California San Diego, San Diego, CA USA; 5 Yale School of Medicine, New Haven, CT USA

**Keywords:** Diabetes, Native Hawaiian Health, Polygenic Risk Score, Stroke

## Abstract

**Purpose:**

Diabetes is a highly heritable risk factor for stroke, with many known genetic variants. The role of these genetic variants in cerebrovascular disease has not been studied in Kānaka Maoli (Native Hawaiian) populations. We aimed to determine if genetic predisposition to diabetes is associated with a higher risk of stroke in a Kānaka Maoli population.

**Methods:**

We conducted a genetic association case/control study using data from Kānaka Maoli participants in the Population Architecture using Genomics and Epidemiology (PAGE) study. Stroke cases were identified through health questionnaires and matched by age and sex at a 1:10 ratio. We modeled genetic predisposition to diabetes with a polygenic risk score that included independent genetic variants known to be associated with diabetes at a genome-wide significance level (*p* < 5 × 10^8^). This polygenic risk score served as the independent variable in logistic regression models for both diabetes and stroke.

**Results:**

A total of 440 Kānaka Maoli individuals were included (15% under 50 years old, 85% aged 50–69, 65% female). There were 40 stroke patients and 400 age- and sex-matched control subjects. Genetic predisposition to diabetes was linked to a 23% increased risk of developing diabetes (OR 1.23, CI 1.01–1.51; *p* = 0.04) and a 45% increased risk of stroke (OR 1.45, CI 1.03–2.05; *p* = 0.04).

**Conclusions:**

In a Kānaka Maoli population, genetic predisposition to diabetes is associated with an increased risk of stroke. Future research to identify effect modifiers of this relationship may uncover significant public health strategies.

## Introduction

Although stroke is common worldwide, epidemiological studies have shown that its burden is not evenly spread across racial and ethnic groups [1]. Evidence mainly focuses on disparities faced by Black and Hispanic populations [2]. However, the public health challenges caused by health disparities affect many more groups that have faced social, economic, and environmental disadvantages based on race or ethnicity.

Hawaiʻi stands out for its unique population distribution, leading the nation in racial and ethnic diversity [3]. However, diversity alone does not ensure health equity. Studies have shown that people identifying as Kānaka Maoli (Native Hawaiian) or Pacific Islander (NHPI) bear a disproportionate burden of cerebrovascular disease. In a recent comparative study of acute ischemic stroke incidence rates in 4 US states, NHPI individuals had approximately 3.3 times higher age- and sex-standardized incidence rates compared to non-Hispanic Whites [4]. Previous research found that among stroke patients admitted to Hawaiʻi’s major tertiary stroke referral center, NHPI patients who presented with ischemic stroke were nearly a decade younger and more likely to have diabetes, hypertension, and obesity. Diabetes in particular demonstrated the strongest association among risk factors for stroke risk in NHPI individuals compared to Whites, with an odds ratio of 2.74 (95% CI 1.87–4.00.87.00) in multivariable regression analysis [2]. In a Northern California study, NHPI had the highest diabetes prevalence among all racial/ethnic groups studied, reaching an age-adjusted prevalence of 34.5% (95% CI 33.1%−35.9%), compared to non-Hispanic Whites (12.8%), Asians (15.6%−31.9%), Blacks (24.9%), and Hispanics (25.3%) [5].

The direction of causation between T2D and stroke in this population is difficult to determine; however, there may be shared genetic factors contributing to a common pathophysiology, indicating that genetic susceptibility to T2D may serve as a potential predictor of stroke risk. While social determinants of health must be addressed to create more equitable stroke health systems in Hawaiʻi [6], research involving data on genotypes can explore disease risks and responses to treatments related to genetic and epigenetic variation. Race, ethnicity, and Indigeneity convey different information from genetic ancestry. Still, these constructs are often closely connected, so informed use of genetic analyses can help efforts to reduce health disparities among racially identified groups [7]. Early attempts at establishing population-specific trends in precision medicine face challenges due to non-standardized and aggregated data collection methods [8]. To begin understanding whether there is shared genetic susceptibility between T2D and stroke among Hawaiʻi’s underserved populations, we aimed to determine whether genetic predisposition to diabetes is linked to stroke in a population identifying as Kānaka Maoli.

## Methods

### Study design and participants

We analyzed data from the Population Architecture using Genomics and Epidemiology (PAGE): Multiethnic Cohort (MEC) study. A description of the MEC study is available elsewhere [9]. All data are publicly accessible through the National Institute of Health database of Genotypes and Phenotypes.

### Case and phenotype variable ascertainment

Histories of stroke, diabetes, hypertension, and myocardial infarction were documented at baseline assessment (see Table 1).

**Table 1:** Demographic and vascular risk characteristics of Kānaka Maoli participants, overall and stratified by stroke status

Variable	All *n* = 440	Stroke *n* = 40	Non-Stroke *n* = 400
Demographics			
Age			
<50	66 (15.0)	6 (15.0)	60 (15.0)
50–54	110 (25.0)	10 (25.0)	100 (25.0)
55–59	66 (15.0)	6 (15.0)	60 (15.0)
60–64	99 (22.5)	9 (22.5)	90 (22.5)
65–69	99 (22.5)	9 (22.5)	90 (22.5)
Female sex	286 (65.0)	26 (65.0)	260 (65.0)
Vascular risk factors			
Hypertension	196 (44.5)	31 (77.5)	165 (41.3)
Diabetes	73 (16.6)	14 (35.0)	59 (14.8)
Myocardial Infarction	33 (7.5)	8 (20.0)	25 (6.3)

### Genetic data

Blood samples were collected from MEC participants with common cancers, and later, prospective collection of biospecimens from a large portion of the cohort was also conducted [10]. Genotyping was performed using the AB OpenArray platform at the Cancer Research Center of Hawaiʻi and the TaqMan platform at the University of Southern California. A subcohort of individuals were genotyped on the MEGA genotyping array as part of the PAGE consortium [11, 12]. Standard quality control procedures, followed by imputation of unobserved single-nucleotide variants (SNVs) using reference panels from the 1000 Genomes project, were applied separately for each study [13–15]. Principal component analysis was used to control for population structure [16].

### Exposure ascertainment

The exposure of interest was genetic predisposition to diabetes, modeled through a polygenic risk score (PRS) to account for hundreds of SNVs associated with diabetes. The PRS was constructed using 379 common and low-frequency, genome-wide significant, independent (r2 < 0.1) SNVs derived from a large GWAS of diabetes [17]. This GWAS was selected due to the established validity of loci identified, which have been consistently replicated across populations, and its targeted approach using the custom Metabochip array to provide high-quality genotyping of previously established loci. Particularly as ancestry-specific GWAS data are unavailable in Kānaka Maoli populations, this approach prioritized PRS construction from a validated and well-characterized starting point. For each participant, the PRS was calculated as the weighted sum of 740 risk alleles. Effect sizes (beta coefficients) from the discovery GWAS were used as weights.

### Statistical analysis

Regression analyses were used to model the change in stroke risk as a function of the PRS for diabetes. PRSs were converted to a standard normal distribution and entered as a continuous variable in the regression models. This approach allows the beta for the PRS to be interpreted as the change in stroke risk per 1 standard deviation increase in the PRS. Regression models were adjusted for 6 principal components, age, sex, hypertension, and myocardial infarction. Genetic and statistical analyses were performed in PLINK (version 1.9) and R (version 3.6.1), respectively.

## Results

A total of 440 Kānaka Maoli participants were included in the study (see Table 1), comprising 40 participants with a reported history of stroke and 400 without such a history. These subjects were matched by age and sex. Regression analyses revealed a significant association between the PRS for diabetes and a reported history of diabetes (OR 1.23, CI 1.01–1.51, *p* = 0.04), as well as stroke (OR 1.45, CI 1.03–2.05, *p* = 0.04) (see Table 2). Model 1 adjusted for principal components 1 and 2. Model 2 adjusted for age, sex, and principal components 1 and 2 (see Table 3).

**Table 2:** Logistic regression analysis of polygenic risk score (PRS) for diabetes and outcomes in Kānaka Maoli participants

	OR (95%CI)	*P*
Diabetes ~ Diabetes PRS	1.23 (1.01–1.51)	0.043
Stroke ~ Diabetes PRS	1.45 (1.03–2.05)	0.038

**Table 3:** Adjusted models for diabetes polygenic risk score PRS and outcomes. Model 1 adjusted for principal components 1 and 2. Model 2 adjusted for age, sex, and principal components 1 and 2

	OR (95%CI)	*P*
Diabetes ~ Diabetes PRS		
Model 1	1.23 (1.01–1.51)	0.043
Model 2	1.21 (0.98–1.49)	0.08
Stroke ~ Diabetes PRS		
Model 1	1.45 (1.03–2.05)	0.038
Model 2	1.45 (1.02–2.05)	0.037

## Discussion

We present the findings of a case-control genetic association study that examined the link between genetic predisposition to diabetes and stroke in a Kānaka Maoli population. Our results indicate that individuals with a higher genetic risk for diabetes are more likely to experience stroke.

Diabetes is a known risk factor for stroke, but its contribution to different stroke subtypes varies. In populations like NHPI, which have high stroke rates and often young onset, it is crucial to find ways to improve detection and treatment of modifiable risk factors. Our results emphasize the link between diabetes and stroke in a Kānaka Maoli population and suggest that early prevention, diagnosis, and management of diabetes could be effective strategies for stroke prevention in this group.

It is important to acknowledge that while our findings capture the polygenic component of diabetes-related stroke risk in Kānaka Maoli, it is unknown whether monogenic variants contribute to this risk. To our knowledge, there is no published literature specifically examining the prevalence or enrichment of monogenic stroke or diabetes variants in this population [18–20]. Existing genotype data from the PAGE: MEC subcohort analyzed in this study is insufficient for comprehensive analysis of monogenic variant frequency in Native Hawaiians, as the MEC primarily used SNV arrays rather than whole-genome or whole-exome sequencing [21]. SNV arrays are fundamentally limited in their ability to detect rare pathogenic variants that cause monogenic diseases, as they capture common variants and selected known pathogenic variants but systematically miss population-specific rare variants that characterize monogenic stroke and diabetes genes like NOTCH3, GCK, HNF1A, and HNF4A. Accurate detection of monogenic variants requires sequencing-based approaches, and imputation-based approaches would require population-specific reference panels, which currently do not exist for Native Hawaiians [22–24].

Additionally, in order to ascertain the significance of the relationship between diabetes genetic risk and stroke in this population, testing additional known cerebrovascular risk factors such as hypertension, smoking, and coronary artery disease would be an important future direction of this research. Similar to diabetes, large-scale studies have demonstrated overlap in the genetic architecture of stroke risk and these conditions [25–28] thus evaluating these relationships in the understudied NHPI population presents a natural next step in this work.

Approximately 90% of current large-scale screens of human genetic variation feature individuals of European ancestry [29]. It is important to note that the genetic variants related to diabetes in our constructed PRS were mainly identified in individuals of European ancestry. Because the accuracy of risk prediction using genetic information decreases as genetic differences between the discovery and target populations increase [30], the detection of genetic predisposition to diabetes in our study was likely biased toward underestimating the actual risk. Any genetic screening tool for diabetes based on currently available data may fail to identify actual health risks in understudied populations, including Indigenous peoples of the Pacific. Furthermore, among individuals with recently admixed genomes, genetic ancestries may vary individual to individual and locus to locus in the genome [31]. Kānaka Maoli are the group most likely to report having two or more components of ancestry in the United States census [32], deriving major continental ancestry from Polynesians, and recent admixtures primarily from European and East Asian immigrants within the last 300 years. Accuracy of PRS models for phenotypes including T2DM were recently described in the Kānaka Maoli subpopulation of the MEC, in a study which found that PRS performance was partially affected by individual differences in ancestry proportions—and particularly reduced among a subcohort of individuals with higher Polynesian ancestry. These evaluations suggest that recent continental-level admixture may alleviate, or mask, PRS transferability issues affecting this admixed and understudied population [33]. Thus, local efforts to promote inclusive, community-based, collaborative research aimed at finding disease-associated variants more common in Kānaka Maoli communities are crucial as precision medicine tools continue to be developed based on genetic information.

The success of these efforts depends not only on who is included but also on how research is conducted—specifically, in ways that uphold Indigenous Data Sovereignty. This involves centering Kānaka Maoli priorities, engaging community members in the research process, and ensuring that findings are returned and meaningfully contextualized with the communities from which the data originate. As part of this commitment, we aim to share results in accessible, culturally grounded formats and to support community education that increases understanding of the benefits and risks of genomic research. These practices are essential not only for ethical integrity but also for enhancing the usefulness of precision medicine—making sure that tools are developed through partnership and responsive to both genomic variation and the broader social determinants of health.

In conclusion, we found that a stronger genetic predisposition to diabetes is linked with stroke in a Kānaka Maoli population. Follow-up studies are necessary to identify effect modifiers of this association and to explore potentially rare disease-related variants in understudied populations.

## Data Availability

The data that support the findings of this study are available from the NIH database of Genotypes and Phenotypes (dbGaP).
